# Impact of COVID-19 Pandemic in Antibiotic Consumption in Navarre (Spain): An Interrupted Time Series Analysis

**DOI:** 10.3390/antibiotics12020318

**Published:** 2023-02-03

**Authors:** Natalia Alzueta, Amaya Echeverría, Patricia García, Lorea Sanz, Alberto Gil-Setas, Xabier Beristain, Pablo Aldaz, Javier Garjón

**Affiliations:** 1 Hospital Reina Sofía, Navarre Health Service, 31500 Tudela, Spain; 2 IdiSNA, Health Research Institute of Navarre, 31008 Pamplona, Spain; 3 Subdirectorate of Pharmacy, Navarre Health Service, 31002 Pamplona, Spain; 4 Clinical Microbiology Department, Hospital Universitario de Navarra, Navarre Health Service, 31008 Pamplona, Spain; 5 San Juan Primary Care Center, Navarre Health Service, 31011 Pamplona, Spain

**Keywords:** antibiotic, COVID-19, primary care

## Abstract

On 11 March 2020, the World Health Organization declared coronavirus disease 19 (COVID-19) a global pandemic. This exceptional situation changed the world not only in terms of mortality and morbidity, but also in terms of epidemiology and health system resources consumption. The objective of this work was to analyze the consumption of antibiotics during the period around the pandemic in our region. A drug utilization study was performed comparing the antibiotic consumption in the community during the years 2018, 2019, 2020, and 2021. Quarterly antibiotic use (defined daily doses (DDD) per 1000 inhabitants per day (DID)) and number of patients treated were the outcomes. Interrupted time series regression analysis was performed to estimate the statistical significance of the change in level of consumption before and after the COVID-19 pandemic. The drop of global antibiotic consumption was statistically significant, both in number of patients and in DID when analyzing pre-pandemic period versus pandemic period. The use of strategic antibiotics for respiratory infections such as amoxicillin, amoxicillin-clavulanic acid, and levofloxacin also decreased significantly. Seasonal pattern of use of antibiotics disappeared due to the global measures imposed over the world to work against COVID-19.

## 1. Introduction

The emergence and spread of infections caused by bacteria that are resistant to antibiotics is one of the most serious threats facing public health and one of the most important challenges for medicine [1].

The increase in resistance is due to various factors such as the inappropriate and indiscriminate use of antibiotics, which causes a great clinical, epidemiological, and microbiological impact.

The increasing concern about bacterial resistance has made the rational prescription of antibiotics even more urgent. This situation led to the creation of the Joint Action Antimicrobial Resistance and Healthcare-Associated Infectious (EU-JAMRAI) in order to implement effective One Health policies to combat Antimicrobial Resistance and reduce Healthcare-Associated Infections [2]. In Spain, this was turned into a National Plan against Antibiotic Resistance which is focused on monitoring the consumption of antibiotics and raising public awareness [3]. One of the objectives of the National Plan against Antibiotic Resistance is the implementation of antibiotic stewardship programs, both in hospitals and in primary care. These programs work in order to optimize the prescription of antibiotics to improve the prognosis of patients who need them, minimize adverse effects, control the emergence of resistance, and guarantee the use of cost-effective treatments [3].

In Spain, more than 90% of the antibiotics are prescribed in primary care setting. Two-thirds of patients treated for infectious diseases receive antibiotic therapy [4]. This leads to 25–30% of the population receiving antibiotics annually [5,6]. Therefore, antibiotic stewardship programs in primary care are a priority [3,7,8].

The actions of Spanish National Plan against Antibiotic Resistance in primary care include surveillance of antibiotic consumption and antimicrobial resistance, training and information and recommendations for healthcare professionals. The main objectives of this plan are to reduce the use of antibiotics for systemic use, to prioritize the use of narrow-spectrum antibiotics (penicillin, amoxicillin, fosfomycin), and to reduce the use of quinolones, amoxicillin-clavulanic acid, macrolides, and third-generation cephalosporins. The improvement in the use of quinolones and amoxicillin-clavulanic acid deserves maximum priority due to its ecological impact, its strategic relevance, and its high volume of prescription [3]. In our region, antibiotic stewardship in primary health care is gradually being implemented [9].

Suddenly, on 11 March 2020 the World Health Organization (WHO) declared coronavirus disease 19 (COVID-19) a global pandemic [10]. A novel coronavirus, Severe Acute Respiratory Syndrome Coronavirus 2 (SARS-CoV-2), responsible for COVID-19, had rapidly spread to become the focus of healthcare systems worldwide [11]. Its highly contagious nature with more than 640 million people infected and more than 6.6 million deaths by November 2022 has led to its prioritization as a public health issue [12]. This exceptional situation changed the world not only in terms of mortality and morbidity, but also in terms of epidemiology and health system resources consumption. 

Since the beginning of the pandemic, most researchers have focused on developing treatment strategies and measures to work against COVID-19. The worldwide situation changed and, in order to prevent the infection, several non-pharmacologic strategies were implemented, such as lockdowns, including physical distancing, protective masks, and promotion of hand hygiene, among others [13,14].

During the first wave of the COVID-19 pandemic, Spain was one of the most heavily affected countries. It has been estimated there was a 12% excess in deaths in Spain in 2020 due to COVID-19, with figures in the region of Navarre of the same magnitude [15]. The government response at the beginning of this pandemic was a national lockdown from 14 March to 21 June 2020. Several measures were adopted so that people were urged to stay at home as much as possible, hand hygiene measures were encouraged, mandatory use of face masks was established, as well as social distance and restriction of travels, meetings, and public activities. Schools, restaurants, bars, theatres, and sport facilities were closed and visits to nursing homes were not allowed. Throughout 2020 and 2021, six epidemic waves affected Spain. Navarre followed the same epidemiologic pattern [16]. 

In Spain, the great majority of studies involving antibiotic consumption during the COVID-19 pandemic have been carried out in a hospital setting. So far, only one study has been carried out in Primary Care. This work is a cross-sectional study comparing antibiotic use in the community in the first and second quarters of 2019 and 2020 in a southern region of the country (Andalusia, Spain) [17].

The objective of our work was to analyze the consumption of antibiotics and the evolution during the years 2018, 2019, 2020 (COVID-19 pandemic), and 2021 in our region (Navarre, Spain).

## 2. Materials and Methods

### 2.1. Design

A drug utilization study was performed comparing the antibiotic consumption in the community during the years 2018, 2019 (pre-COVID pandemic), 2020 (COVID period), and 2021 (post-COVID pandemic). Outpatient antibiotic use was assessed for the public primary healthcare system of Navarre (Spain) that provides drugs benefits to almost the whole population, of around 653,000 inhabitants.

Quarterly antibiotic use was assessed for overall antibacterial for systemic use (WHO’s ATC Classification System group J01) [18]. We analyzed seven antibiotic groups: J01A (tetracyclines), J01C (penicillins), J01D (other β-lactam antibacterials), J01E (sul-phonamides and trimethoprim), J01F (macrolides, lincosamides, and streptogramins), J01M (quinolones), and J01X (other antibacterials such as fosfomycin).

According to the Spanish Plan against Antibiotic Resistance, we also studied five antibiotics considered critical for an appropriated use of antibacterial agents: amoxicillin, amoxicillin-clavulanic acid, cefuroxime, azithromycin, and levofloxacin [3].

### 2.2. Data Sources and Outcomes

The number of patients and DDD were obtained through databases from computerized pharmacy records of dispensed drugs reimbursed by the Navarre Health Service. Authors accessed aggregated data, meaning that individual patients could not be identified.

Antimicrobial consumption rates were defined in daily doses (DDD) per 1000 inhabitants/days (DID). Patients with prescriptions from private doctors were not included.

Defined daily doses (DDD) per 1000 inhabitants per day (DID) and the number of patients treated were outcomes.

### 2.3. Statistical Analysis

A descriptive analysis of the data was performed. We calculate absolute variation of strategic antibiotics consumption between each quarter of the years 2019 and 2021.

Interrupted time series regression analysis was carried out to estimate the statistical significance of the change in level of consumption before and after the start of COVID-19 pandemic. The time points were the quarters of 2018, 2019, 2020, and 2021. The second quarter 2020 was considered the first period of pandemic. The Durbin–Watson statistic, with a level of significance of 5%, was used to assess autocorrelation. Since evidence of autocorrelation was not detected, the analysis was done without adjustment for autocorrelation. We used quarter as the dummy variable to adjust for seasonality [19,20].

IBM SPSS Statistics v. 25.0 software was used.

### 2.4. Ethics Statement

This manuscript is the result of the work of medical and pharmaceutical staff in the exercise of their functions of evaluation of the quality of prescription and promotion of drug safe use. It did not imply access to the data by people other than those who usually handle it, and data used were anonymized. By Spanish law, the aggregate information resulting from processing prescriptions of the National Health System is of public domain and its management is competence of regional health services [21]. The request and handling of aggregate data guarantees the confidentiality of the same, given that no references are made to personal data, only to aggregate data by region. The information has been provided by Navarre Health Service. Therefore, the authorization of a Drug Research Ethics Committee was not required.

## 3. Results

**Figure 1:**
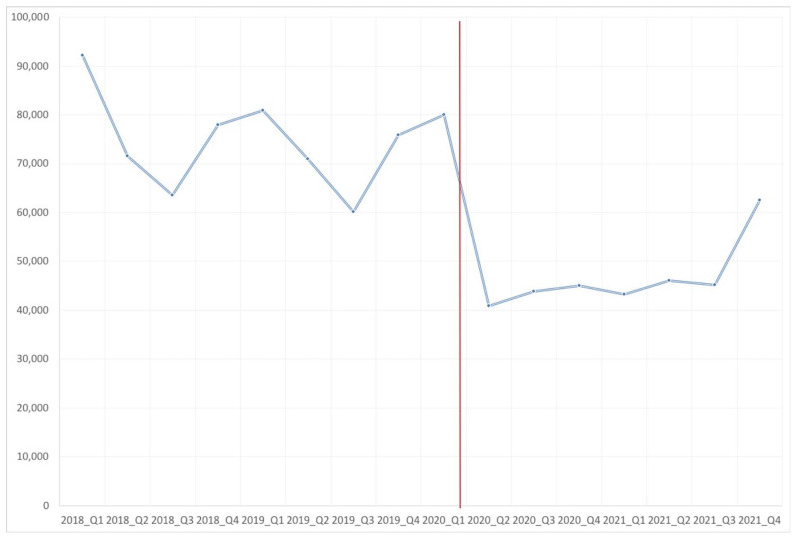
Number of patients treated with systemic antibiotics between the years 2018 and 2021 (year-quarter).

The number of patients treated with a systemic antibiotic dropped drastically between the first and the second quarter of 2020 due to the COVID-19 pandemic. This reduction was maintained for 2020 and 2021 and started to increase in the fourth quarter of 2021 (Figure 1).

**Figure 2:**
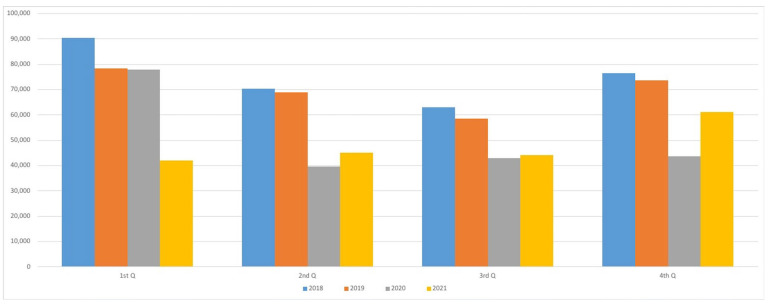
Number of patients treated with an antibiotic between the years 2018 and 2021 (year-quarter).

Typically, antibiotics consumption shows a seasonal pattern, with a maximum in the first and fourth quarter of the year due to the high incidence of respiratory tract infections in winter. However, according to our data, this seasonality disappeared during the pandemic, especially in 2020 (Figure 2). A drastic reduction was observed at the beginning of the second quarter of 2020, coinciding with the Spanish lockdown that was in force from 14 March to 21 June 2020.

The drop of global antibiotic consumption was statistically significant in number of patients (*p* = 0.007) and in DID (*p* = 0.007) when analyzing the pre-pandemic period versus the pandemic period (Table 1).

When analyzing evolution of the consumption (Table 1), we observed that since the beginning of the pandemic in March 2020, consumption of most evaluated antibiotics dropped; this happened in the second, third, and fourth quarter of the year and it was also observed for the global amount of antibiotics used. Furthermore, it was observed that in the fourth quarter of 2021, global consumption of antibiotics started to rise, although it did not reach 2018 levels. 

In addition, antibiotics whose decline was statistically significant were penicillin (J01C), macrolides (J01F), and quinolones (J01M), with all of them considered of critical importance according to the Spanish National Plan against Antibiotic Resistance (Table 1).

**Figure 3:**
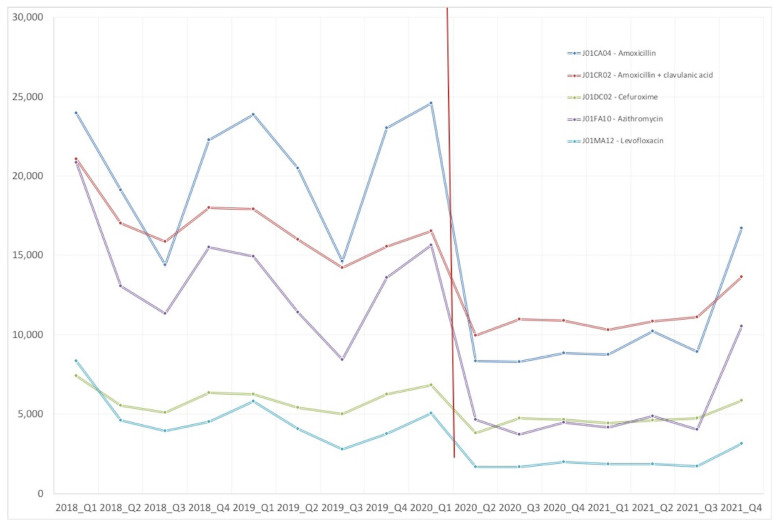
Patients treated with considered critical antibiotics among the years 2018 and 2021.

If we analyze the evolution of specific strategic antibiotics for respiratory tract infections, as shown in Table 2, the greatest reductions, occurring between the first quarter of 2019 and the first quarter of 2021, were especially related to the consumption of amoxicillin, amoxicillin-clavulanic acid, and levofloxacin. These reductions were observed both in the number of patients treated and in DID. These antibiotics are considered critical for an appropriate use according to the Spanish National Plan against Antibiotic Resistance [3]. In Figure 3, we can see the drastic descent in the number of patients treated with critical antibiotics, descending more than 50% in cases such as amoxicillin and azithromycin.

## 4. Discussion

The increase in antibiotic-resistant bacteria and the scarce prospects for the development of new antimicrobials have led to the implementation of antimicrobial stewardship interventions, with numerous actions carried out both at hospitals and in the community. Antibiotic-resistant bacteria are a huge problem in hospitals; however, the global control of bacterial resistance can only be achieved by improving the use of antimicrobials in the community [8]. That is why, in our region, antibiotic stewardship in Primary Health Care started in 2018 and has been progressively implemented [9].

In March 2020, with the arrival of the COVID-19 pandemic, caused by SARS-CoV-2, many aspects of the approach of the respiratory tract infections, including the use of antibiotics, were affected. It is known that the use of antibiotics in hospitals increased during the pandemic [14]. However, in a primary care setting, the latest data from the European Centre for Disease Prevention and Control (ECDC) show a decrease in the total antibiotic consumption in humans by more than 15% between 2019 and 2020 [22,23].

Our study shows that the antibiotic use in primary care setting decreased in the COVID period compared to the pre-COVID period. If we compare consumption (DID) in the first quarter of 2020 and 2019 the reduction was 3%, however, if we compare the second quarter of 2020 and 2019 a decrease of 39% is observed. During this period, the lockdown was more extensive. That is why this fact could have helped to reduce the transmission of other respiratory infections where antibiotics are prescribed, and less accessibility to health centers may have helped to reduce unnecessary antibiotic prescriptions. This resulted in fewer antibiotic prescriptions for mild and self-limiting infections. Other non-pharmacological measures, such as face masks, hand hygiene measures, and physical distance, could have helped to reduce the transmission of respiratory infections [14].

Our results were similar to those reported in another Spanish study carried out by Peñalva et al. [17]. This work is a cross-sectional study comparing antibiotic use in the community in the first and second quarters of 2019 and the same quarters in 2020 (COVID-19 period) in Andalusia (Spain). They showed a 36.8% reduction in the global use of antibiotics (DID) when comparing the second quarter in the years 2020 and 2019. 

Similar to Peñalva et al., we observed that since the beginning of the pandemic in March 2020, the use of most of the evaluated antibiotics dropped, as seen in the quarterly time-series analysis [17]. Our study shows the data of antibiotic consumption among the years 2018 and 2021, adding more information on the evolution of antibiotic consumption to that already included in Peñalva’s work in order to analyze if the use of antibiotics only was reduced during the pandemic or it was maintained during 2021. Our data show that in the fourth quarter of 2021, global consumption of antibiotics started to raise, although it did not reach 2018 levels. Moreover, we included data of the antibiotics considered of critical importance according to the Spanish National Plan against Antibiotic Resistance, whose use should be reduced.

Among the priority objectives of the Spanish National Plan against Antibiotic Resistance in primary care setting is the improvement in the use of quinolones, amoxicillin-clavulanic acid, macrolides, and third-generation cephalosporins. The improvement in the use of quinolones and amoxicillin-clavulanic acid deserves maximum priority due to its ecological impact, its strategic relevance, and its high volume of prescription [3]. In fact, according to some studies, resistance to quinolones and beta-lactams represented more than 70% of deaths attributable to bacterial antimicrobial resistance in 2019 in the world [24]. If we compare the consumption of levofloxacin and amoxicillin-clavulanic acid in the second quarter of 2019 to the same period in 2021, a reduction of 52% and 35%, respectively, is observed. Despite the pandemic having influenced the reduction in the use of antibiotics, this reduction has maintained over time.

When evaluating the use of strategic antibiotics for respiratory tract infections, our results are of a similar magnitude to those observed by Peñalva et al., taking into account the fact that their results were obtained between 2019 and 2020, while ours refers to the difference between 2019 and 2021 [17].

At the beginning of the COVID-19 pandemic, azithromycin in combination with hydroxychloroquine was proposed as a potential therapy for the treatment of SARS-CoV-2 infection. A low-quality study with low sample size and many methodological limitations concluded that this combination reduced the nasal viral load in patients with COVID-19 [25]. However, this treatment option was ultimately rejected because, in hospitalized patients, azithromycin did not improve survival or other prespecified clinical outcomes such as progression to severe course or intensive care unit admission [26]. If we compare the second quarter of 2019 and 2021, we observe a reduction of 54% in DID of azithromycin. It is important to remark that antibiotics are ineffective for viral syndromes, including COVID-19. These observations reinforce the importance of the antibiotic stewardship programs improving appropriate antibiotic prescribing and avoiding unnecessary antibiotic use for viral infections [27].

According to Gagliotti et al. [28], the reduction in antibiotic consumption suggests that the lockdown had a significant impact on the transmission dynamics of COVID-19 and other respiratory tract infections. Waiting strategies and the reduction of ambulatory visits may contribute to this reduction.

Our data were also compared with those published by the Spanish Agency of Medicines on a national level to verify whether or not there was support for the hypothesis regarding the reduction of use of antibiotics, secondary to the decrease in respiratory infections, as occurred in the Navarre health area. The decrease in the use of antibiotics is clear both on a national and local level [29]. An exception for this find is the consumption of sulfonamides and trimethoprim (J01E), which remained stable during 2020 and 2021. This is probably due to the fact that these antibiotics are mainly used for other infections that are not respiratory, such as skin and soft tissue infections or urinary tract infections. The same occurred with group J01X (other antibacterials), which includes fosfomycin and nitrofurantoin used for urinary tract infections. This is consistent with the stability of the incidence of urinary infections and subsidiary pathologies treated with these therapeutic groups. Seasonal trends of use of antibiotics are another aspect that has been evaluated in this work. Respiratory tract infections are known to have a high incidence of infection during winter, especially in temperate regions [30]. The maximum use of antibiotics is typically observed in the first and fourth quarter of the year. However, this pattern has disappeared during the pandemic, especially in 2020. The COVID-19 lockdown has changed the panorama of community-acquired infections, with effects on the total consumption of antibiotics. The reasons for this change are the confinement, the mandatory use of face masks, social distance measures, and hygiene measures [14]. These strategies have proven to be effective, and they could be applied in order to prevent respiratory tract infections and to reduce the antibiotic consumption in different risk groups such as elderly people or COPD patients. Thus, the clinical implications of this work are that reinforcement of physical and hygienic measures must play an important role, especially in vulnerable populations, in the policies of prudent use of antimicrobials.

The major strength of this work is the comprehensiveness of data, obtained from computerized pharmacy records of reimbursed and dispensed drugs of the Navarre Health Service, which comprises a population of approximately 653,000 inhabitants. There is a lack of studies in primary care because the majority of the COVID-19 research has been conducted in hospital settings. However, it is necessary to generate more knowledge in primary care settings. 

The use of DDD as a unit of measure of antibiotics consumption has several limitations. DDD is not always the dose actually used, especially in children. The duration of treatment of different antibiotics, even for the same indication, can be different. Nevertheless, our results for the outcomes DID and number of patients are largely consistent.

A limitation of this work is that patients with prescriptions from private doctors were not included.

Future directions:

The pandemic has shown that epidemiology of infectious diseases can change abruptly, as can the policies of infection control (use of face masks, lockdowns, and restrictions on social contact). The development of new vaccines and treatments has speed up. Therefore, the monitoring of use of antimicrobials must be continuous, with information about the indications treated and linked with the resistance monitoring. In addition, antibiotic stewardship programs must be flexible enough to adapt to an unstable environment.

## 5. Conclusions

During the COVID-19 pandemic period, the number of outpatients with antibiotic prescriptions decreased substantially in our region, with the sharpest decline occurring in the second quarter of 2020. 

Antibiotics whose decline was statistically significant were betalactams, macrolides, and quinolones, which were considered of critical importance according to the Spanish National Plan against Antibiotic Resistance.

Seasonal patterns of respiratory tract infections have disappeared during the pandemic due to the lockdown, the mandatory use of face masks, social distance measures, and hygiene measures, among others. Some of these strategies, such as the use of face masks and hands hygiene measures, have proven to be effective in order to prevent respiratory tract infections and to reduce the antibiotic consumption. For this reason, they should be maintained, especially in vulnerable populations.

It is important to continue with antibiotic stewardship programs in primary care in order to optimize the use of antibiotics in the community.

## Figures and Tables

**Table 1:** Evolution of the consumption of antibacterials for systemic use (ATC group J01) in the community in Navarre per quarters (DID and number of patients).

	1st Quarter				2nd Quarter				3rd Quarter				4th Quarter				
	2018	2019	2020	2021	2018	2019	2020	2021	2018	2019	2020	2021	2018	2019	2020	2021	*p* *
**Defined daily doses per 1000 inhabitants per day (DID)**																	
J01A—Tetracyclines	1.04	1.15	1.24	1.60	0.96	1.02	0.93	1.22	0.66	0.77	0.79	0.83	0.99	1.17	1.25	1.24	0.796
J01C—Betalactams. Penicillins	11.00	10.52	10.04	4.97	8.85	9.16	4.86	5.26	7.55	7.52	5.06	5.14	9.50	9.28	5.09	7.23	0.010
J01D—Other betalactam antibacterials	2.66	2.31	2.46	1.62	1.99	1.98	1.45	1.68	1.80	1.83	1.69	1.72	2.19	2.24	1.69	2.09	0.051
J01E—Sulfonamides and trimethoprim	0.31	0.33	0.41	0.40	0.31	0.36	0.36	0.40	0.29	0.39	0.36	0.41	0.30	0.40	0.38	0.39	0.962
J01F—Macrolides. Lincosamides and streptogramins	2.74	2.19	2.22	0.98	1.92	1.72	0.94	1.03	1.62	1.38	0.82	0.87	2.09	1.92	0.95	1.50	0.011
J01M—Quinolones	2.99	2.12	1.66	0.92	1.95	1.60	0.89	0.89	1.78	1.29	0.92	0.91	1.79	1.41	1.00	1.20	0.011
J01X—Other antibacterials	0.38	0.42	0.45	0.46	0.40	0.43	0.43	0.45	0.43	0.48	0.48	0.48	0.42	0.47	0.46	0.47	0.078
TOTAL	21.13	19.05	18.48	10.95	16.37	16.27	9.85	10.93	14.13	13.65	10.11	10.35	17.29	16.90	10.83	14.12	0.007
**Number of patients**																	
J01A—Tetracyclines	1325	1436	1550	1878	1364	1404	1277	1680	975	1075	1093	1151	1284	1465	1573	1516	0.643
J01C—Betalactams. Penicillins	45,217	43,171	42,977	19,741	37,023	38,970	19,170	21,891	30,881	30,950	20,373	21,317	41,118	41,063	20,391	31,333	0.008
J01D—Other betalactam antibacterials	11,008	9554	10,427	6839	8350	8333	6112	7185	7706	7738	7333	7395	9424	9581	7365	9025	0.056
J01E—Sulfonamides and trimethoprim	833	941	1280	1167	932	1087	1100	1237	890	1234	1109	1275	910	1298	1166	1243	0.817
J01F—Macrolides. Lincosamides and streptogramins	23,083	17,043	17,527	5602	15,184	13,231	5843	6430	13,091	10,107	5042	5402	17,593	15,518	5855	12,040	0.013
J01M—Quinolones	16,949	11,645	9241	4707	11,010	8814	4737	4667	10,090	7155	4865	4804	10,289	7948	5258	6641	0.010
J01X—Other antibacterials	7940	8773	9723	9888	8413	9206	9196	9693	9399	10,397	10,730	10,618	9256	10,298	10,287	10,131	0.019
**TOTAL**	92,186	80,882	79,952	43,256	71,561	71,035	40,809	46,108	63,529	60,179	43,869	45,160	77,921	75,839	44,964	62,580	0.007

* Comparing periods pre-pandemic and pandemic.

**Table 2:** Evolution of the consumption of considered strategic antibacterials for respiratory tract infections in the community in Navarre per quarters (DID and patients treated).

		1st Quarter			2nd Quarter			3rd Quarter			4th Quarter			*p* *
		2019	2021	Variation	2019	2021	Variation	2019	2021	Variation	2019	2021	Variation	
J01CA04—Amoxicillin	DID	5.044	1.989	−61%	4.247	2.189	−48%	3.220	1.947	−40%	4.668	3.375	−28%	0.017
	Patients	23,571	8636	−63%	20,277	10,152	−50%	14,637	8889	−39%	22,833	16,544	−28%	0.013
J01CR02—Amoxicillin + clavulanic acid	DID	4.997	2.737	−45%	4.377	2.826	−35%	3.875	2.903	−25%	4.072	3.525	−13%	0.003
	Patients	17,761	10,264	−42%	15,867	10,851	−32%	14,274	11,205	−22%	15,520	13,678	−12%	0.003
J01DC02—Cefuroxime	DID	1.556	1.117	−28%	1.324	1.160	−12%	1.249	1.176	−6%	1.522	1.434	−6%	0.048
	Patients	6093	4389	−28%	5249	4604	−12%	4924	4732	−4%	6111	5871	−4%	0.042
J01FA10—Azithromycin	DID	1.525	0.513	−66%	1.165	0.533	−54%	0.894	0.474	−47%	1.338	1.054	−21%	0.044
	Patients	14,780	4158	−72%	11,336	4856	−57%	8388	4041	−52%	13,409	10,446	−22%	0.017
J01MA12—Levofloxacin	DID	1.099	0.384	−65%	0.765	0.371	−52%	0.550	0.367	−33%	0.688	0.587	−15%	0.022
	Patients	5787	1864	−68%	4068	1861	−54%	2799	1751	−37%	3757	3130	−17%	0.027

* Comparing periods pre-pandemic and pandemic. DID: Defined daily doses per 1000 inhabitants per day.

## Data Availability

Data that support the findings of this study are available on request from the corresponding author.
